# Severe congenital neutropenia caused by the *ELANE* gene mutation in a Vietnamese boy with misdiagnosis of tuberculosis and autoimmune neutropenia: a case report

**DOI:** 10.1186/s12878-015-0020-x

**Published:** 2015-01-24

**Authors:** Quang Van Vu, Taizo Wada, Tham Thi Tran, Duc Ngoc Ngo, Thuc Van Dinh, Cuong Hung Nguyen, Huong Thi Minh Le, Akihiro Yachie, Sang Ngoc Nguyen

**Affiliations:** Department of Pediatrics, Haiphong University of Medicine and Pharmacy, 72 A Nguyen Binh Khiem, Ngo Quyen, Haiphong Vietnam; Center for Clinical Laboratory Medicine, Haiphong University of Medicine and Pharmacy, Haiphong, Vietnam; Haiphong Children Hospital, Haiphong, Vietnam; National Hospital of Pediatrics, Hanoi, Vietnam; Department of Pediatrics, Institute of Medical, Pharmaceutical and Health Science, Kanazawa University, Kanazawa, Japan

**Keywords:** Congenital severe neutropenia, ELANE, Severe bacterial infection, Autoimmune neutropenia

## Abstract

**Background:**

Severe congenital neutropenia (SCN) is an immunodeficiency disease characterized low blood neutrophil counts, early bacterial infections, and risk of leukaemia development. Heterozygous mutations in the *ELANE* gene coding neutrophil elastase are associated with SCN. Patients with SCN suffer from recurrent bacterial infections and often succumb them. To our knowledge, this is the first report of SCN from Vietnam.

**Case presentation:**

A 6-year-old boy was admitted due to severe bacterial infection and severe neutropenia. He had recurrent infections from 8 months of age, and was misdiagnosed with tuberculosis and and autoimmune neutropenia in infancy at 21 and 41 months of age, respectively. His medical report has showed severe neutropenia for many times. In direct DNA sequencing analysis, we found an *ELANE* gene mutation (R81P), which had been confirmed to cause SCN.

**Conclusion:**

The missed and delayed diagnosis may be attributable to insufficient awareness of this rare disease on the background of frequent infections even in the immunocompetent pediatric population in Vietnam. Our results indicate further evidence for the role of *ELANE* in SCN.

## Background

Severe chronic neutropenia is a heterogeneous group of rare disorders due to intrinsic defect of myeloid cell proliferation and maturation [1]. Typically, severe congenital neutropenia (SCN) is defined by extremely low absolute neutrophil count (ANC) (<0.5 × 10^9^/L for at least three months) and recurrent life-threatening bacterial infections. The incidence of SCN is estimated to be 1 in 200,000 individuals [2]. Among several associated genetic mutations, heterogeneous mutations of the *ELANE* gene coding for neutrophil elastase (NE) have been associated with both SCN and cyclic neutropenia (CN), and it is known to be correlated with more severe neutropenia and serious clinical manifestation in SCN [1,3,4].

There have few reports of SCN from developing countries. Moreover, clinical signs of this rare condition are frequently overlapped with other infectious diseases, sometimes resulting delayed or missed diagnosis. Herein, we report for the first time a Vietnamese boy with SCN, confirmed by mutation analysis of the *ELANE* gene in an attempt to improve the diagnosis and management of SCN.

## Case presentation

**Figure 1:**
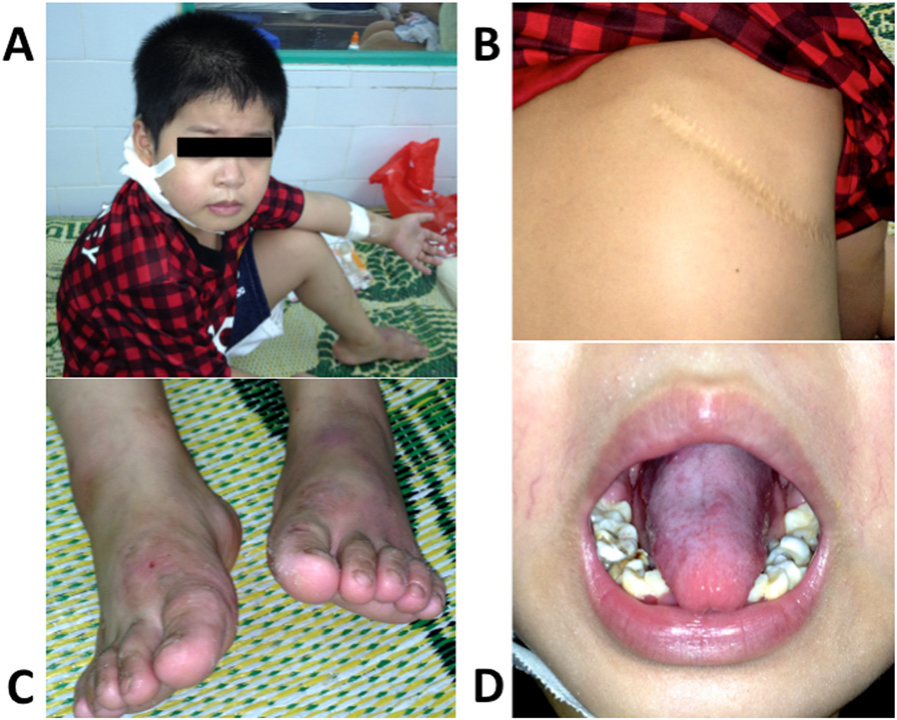
**A number of lesions caused by infection. (A)** cutaneous abscess behind the right ear; **(B)** right parietal lobectomy; **(C)** fungal infection; **(D)** mouth ulcer.

A 6-year-old boy was hospitalized at Haiphong Children Hospital because of phlegmon behind his right ear and high fiver (40°C). Physical examination exhibited consciousness, pale skin, cutaneous abscess behind right ear, pustulosis on skin, foot fungus, and mouth ulcer (Figure 1A,C,D). Laboratory studies revealed severe neutropenia (white blood cells, 6.9 × 10^9^/L; neutrophils, 0.2 × 10^9^/L; lymphocytes, 2.67 × 10^9^/L; monocytes, 3.38 × 10^9^/L). A gram-stain and culture of abscess fluid showed S. aureus, which was susceptible to vacomycin and amikacin. Bone marrow aspirate exhibited normal myeloid cells, reduced granulocyte cell line, but no malignant cells. Serum titers of IgG, IgM, IgA and IgE and percentage of CD4^+^, CD8^+^ T cells were normal. Tests of HIV, HBV, HCV, EBV, and CMV were negative. The patient was treated with antibiotics according to antimicrobial susceptibility testing, abscess incision and drainage, and G-CSF. Two weeks later, the patient was discharged from hospital.

Because of severe neutropenia and infection, we carefully asked medical history and analysed medical records of the patient. From 8 months of age, the patient had recurrent infections, Staphylococcal pneumonia and Staphylococcal septicemia at 10 and 15 moths of age, respectively. In addition, he had cutaneous abscesses and other respiratory infections frequently. Because his infection status was not fully recovered by using antibiotics, from 23 to 30 months of age, the patient was treated with antituberculosis drugs although there was not any evidence of mycobacterium tuberculosis. At the 41 months of age, the patient had necrotizing pneumonia due to S. aureus leading right parietal lobectomy (Figure 1B). At that time, he was diagnosed with autoimmune neutropenia in infancy (AIN) although anti-neutrophil antibodies were not performed. After using solumedrol, neutropenia and infection statuses got worse. Moreover, patient had severe neutropenia in many times (Table 1).

**Table 1:** **Change in some white blood cell count parameters by age**

**Age (month)**	**WBC (10** ^**9**^ **/L)**	**ANC (10** ^**9**^ **/L)**	**lymphocytes (10** ^**9**^ **/L)**	**Monocytes (10** ^**9**^ **/L)**
10	20,1	0,6	12,4	6,6
17	10,2	0,58	4,8	5,08
18	9,2	0,01	5,9	2,67
23^a^	13,2	3,43	6,6	3,17
41^b^	6,48	1,6	2,6	1,9
41^c^	7,09	0,21	2,26	3,55
42	7,98	0,95	3,19	3,43
43	7,7	0,6	4,46	2,77
45	6,79	0,33	3,87	2,77
48	9,54	1,33	7,5	0,57
48	8,8	5,34	3,69	0,9
49	9,34	0,09	7,65	1,12
57	6,64	0,03	2,98	3,09
58	5,18	0,28	3,26	1,21
70	6,76	0,02	2,88	2,94

WBC, white blood cell count; ANC, absolute neutrophil count; ^a^, starting antituberculosis drugs; ^b^, before giving solumedrol; ^c^, after giving solumedrol.

**Figure 2:**
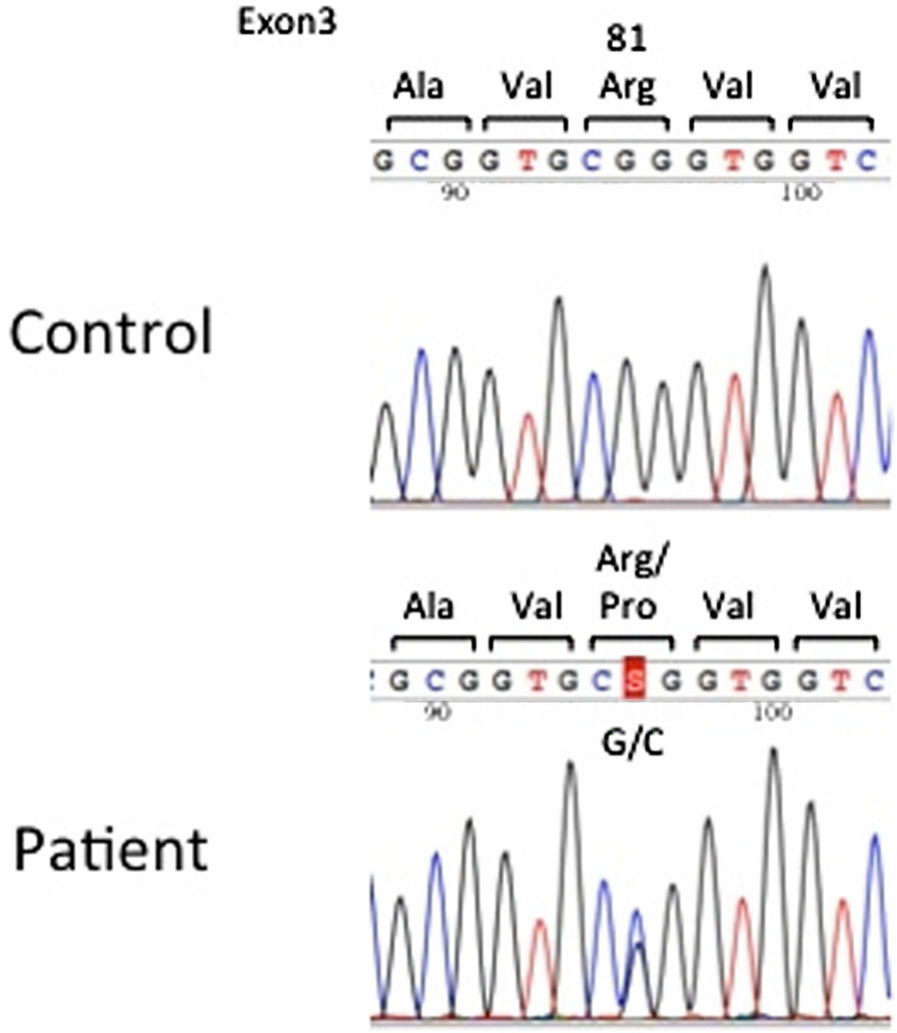
**Sequencing the genomic PCR products, heterozygous missense mutation (R81P)(G changing to C) was identified.**

Considering his medical history, current clinical signs, and laboratory findings, SCN associated with ELANE abnormality was suspected. Direct DNA sequencing analysis demonstrated a heterozygous missense mutation of the 242th base (G to C) in exon 3, resulting change of the 81 codon (Arginine to Proline), which has been reported to cause severe neutropenia (R81P) (Figure 2).

## Conclusions

SCN is a very rare condition that is diagnosed when ANC is less than 0.5 × 10^9^/L for at least three months. Patients with SCN exhibited recurrent life-threatening infections, and a maturation arrest of bone marrow myeloid precursors at the promyelocyte-myelocyte stage of differentiation [4,5]. The boy we herein report showed typical manifestations of SCN including severe neutropenia and recurrent bacterial infections. However, the diagnosis was missed and delayed until six years of age. This issue can be explained by some reasons: Vietnam is a tropical country where infectious diseases are very popular and there is an insufficient awareness of this rare disease on the background of frequent infections even in immunocompetent pediatric population [6]. Moreover, some infections can cause neutropenia such as tuberculosis, Dengue, measles, EV71, HIV, EBV, CMV… Therefore, neutropenia in children really is a clinical challenge for Vietnamese pediatricians especially during the first visit. Because of high prevalence of tuberculosis in Vietnam [7] and non-recovery by routine antibiotics, tuberculosis was suspected in our patient, reflecting an impasse in treatment of infectious diseases without adequate attention to patient’s immune disorders. Differentiating SCN from autoimmune neutropenia (AIN) requires more time for observing the clinical course, but it is important due to specific treatment [8-10]. We were confused because absolute neutrophil count of the patient was sometime elevated. Moreover, AIN is the most common type of chronic neutropenia, the patient was suspected to AIN. However, the more solumedrol he received, the more his neutrophil counts reduced. After treatment with solumedrol, the patient was overwhelming infections, and his ANC was extremely decreased (Table 1). To confirm AIN, detecting anti-neutrophil antibodies is very important but not available in Vietnam now. Carefully analyzing clinical courses of chronic neutropenia patients could contribute to critical clues for different diagnosis. In contract to SCN patients, AIN patients often have mild phenotypes with minor intercurrent infections despite severe neutropenia; their neutrophil counts could vary considerably from day to day and often rise during acute infection without corticosteroid, epinephrine, or G-CSF treatment. Since it is difficult to diagnosis AIN based only on peripheral neutrophil counts and past history, a bone marrow aspiration test is necessary for differential diagnosis [8,10]. Because the patient had severe phenotypes with life-threatening infections, chronic severe neutropenia, and reduced granulocyte cell line on bone marrow aspirate, we suspected SCN. After receiving G-CSF (6 mg/kg/24 h) his neutrophil counts were increased dramatically, excluding mutations of the patient’s G-CSF receptor gene.

To confirm SCN diagnosis, we analyzed the *ELANE* gene mutation firstly because it is the most common gene alteration in SCN (about 50% of SCN and nearly all of CN) [1,5,11]. There have been about 100 reported *ELANE* variants in SCN and CN worldwide. In exon3, we found a heterozygous missense mutation (R81P) (Figure 2). This mutation has been reported to cause severe congenital neutropenia. Because *ELANE* gene has a leader sequence of 29 amino acids, the codon number of the mutation may be also shown as “R52P” [1]. SCN due to *ELANE* mutations causes persistent neutropenia, but surprisingly, ANC of the patient was occasionally elevated. It is difficult to explain, but exceptions may be attributable to infection at the time of blood sampling, which may transiently increase neutrophil production in the patient. Moreover, the disease phenotype is not determined by mutation alone; it can be influenced by genetic, epigenetic, environmental factors in specific time [6,12,13]. To date, this is the first SCN case confirmed by genetic analysis in Vietnam. As the estimated incidence of SCN is 1/200 000 [2], it is suggested that many SCN cases in Vietnam may be undiagnosed. Because G-CSF therapy is available and stem cell transplantation from HLA-identical sibling is the optimal therapeutic modality, early diagnosis is of great importance. National networks and diagnostic guideline for SCN may be helpful for us to improve these issues. Because our patient has an older sibling, we closely follow him with the idea of stem cell transplantation.

In summary, we describe a Vietnamese boy with typical phenotype of SCN but missed and delayed diagnosis. This is the first report of SCN definitively diagnosed by genetic analysis from Vietnam. The missed and delayed diagnosis may be attributable to insufficient awareness of this rare disease on the background of frequent infections even in the immunocompetent pediatric population in Vietnam.

## Consent

Written informed consent was obtained from the patient’s mother for publication of these data and for the accompanying images. A copy of the written consent is available for review by the Editor of this journal.

## Abbreviations

ELANE: ELA2 elastase, neutrophil expressed
SCN: Severe congenital neutropenia
CN: Cyclic neutropenia
AIN: Autoimmune neutropenia in infancy
ANC: Absolute neutrophil count
NE: Neutrophil elastase
G-CSF: Granulocyte colony-stimulating factor
